# Spatial filtering for enhanced high-density surface electromyographic examination of neuromuscular changes and its application to spinal cord injury

**DOI:** 10.1186/s12984-020-00786-z

**Published:** 2020-12-03

**Authors:** Xu Zhang, Xinhui Li, Xiao Tang, Xun Chen, Xiang Chen, Ping Zhou

**Affiliations:** 1 School of Information Science and Technology, University of Science and Technology of China, Hefei, 230027 Anhui China; 2 Institute of Rehabilitation Engineering, University of Rehabilitation, Qingdao, 266024 Shandong China

**Keywords:** Electromyography, Noninvasive diagnosis, Neuromuscular changes, Spatial filtering, Spinal cord injury

## Abstract

**Background:**

Spatial filtering of multi-channel signals is considered to be an effective pre-processing approach for improving signal-to-noise ratio. The use of spatial filtering for preprocessing high-density (HD) surface electromyogram (sEMG) helps to extract critical spatial information, but its application to non-invasive examination of neuromuscular changes have not been well investigated.

**Methods:**

Aimed at evaluating how spatial filtering can facilitate examination of muscle paralysis, three different spatial filtering methods are presented using principle component analysis (PCA) algorithm, non-negative matrix factorization (NMF) algorithm, and both combination, respectively. Their performance was evaluated in terms of diagnostic power, through HD-sEMG clustering index (CI) analysis of neuromuscular changes in paralyzed muscles following spinal cord injury (SCI).

**Results:**

The experimental results showed that: (1) The CI analysis of conventional single-channel sEMG can reveal complex neuromuscular changes in paralyzed muscles following SCI, and its diagnostic power has been confirmed to be characterized by the variance of Z scores; (2) the diagnostic power was highly dependent on the location of sEMG recording channel. Directly averaging the CI diagnostic indicators over channels just reached a medium level of the diagnostic power; (3) the use of either PCA-based or NMF-based filtering method yielded a greater diagnostic power, and their combination could even enhance the diagnostic power significantly.

**Conclusions:**

This study not only presents an essential preprocessing approach for improving diagnostic power of HD-sEMG, but also helps to develop a standard sEMG preprocessing pipeline, thus promoting its widespread application.

## Introduction

Spinal cord injury (SCI) is a leading cause of adult disability worldwide [1]. The disruption of communication between the brain and the spinal cord results in both loss of voluntary movement (i.e., paraplegia) and loss of sensation [1, 2]. However, the effect of a paraplegia on the survival and function of motor unit (MU) in pathological muscles remains unclear. Since the MU is regarded as the basic functional unit and the final pathway of the neuromuscular control system, it is of great importance to identify MU changes induced by specific mechanisms following the SCI [3], which can offer guidance for the design of effective SCI rehabilitation protocols.

In clinical routine, an invasive approach using concentric needle is applied to electrophysiological examination of MU properties [4, 5]. The insertion of the needle, however, has to deal with various issues including the invasive discomfort, a requirement of medical supervision and a risk of infection, limiting its wide applications including long-term monitoring and repetitive investigations [6]. In addition, the subjectivity during diagnostic evaluation based on the experience of the professional clinicians remains the most important factor when it comes to examine neuromuscular changes [7]. As a result, there is a huge demand for an objective, quantitative and noninvasive approach for convenient examination of neuromuscular diseases and injuries.

Surface EMG (sEMG) is a technique to record the electrical activity of muscle using electrodes placed over skin surface. Due to the benefits of non-invasive and low-cost properties, sEMG recording has been used in examining neuromuscular activities [8–10]. However, traditional bipolar electrode configuration of recording sEMG may suffer from difficulties such as noise contamination, cross-talks from a neighboring muscular activities, attenuated motor unit action potential (MUAP) waveforms due to the low-pass filtering effects of skin and subcutaneous body tissues, and the failure to discern or characterize individual MUAP waveforms due to their severe superposition. As a result, conventional sEMG has not been well accepted by clinicians towards diagnostic applications. High-density (HD) electrode grid has been playing an increasingly important role in the collection of sEMG signals. Although any individual surface electrode within the grid works as the regular single-channel, the grid formation warrants collection of important spatial information concerning muscle activation. Therefore, the HD-sEMG measurement is able to better characterize the muscle’s structural and functional heterogeneity, which is regarded as the reflection of activities from different sources such as subcomponent muscles [11–13], muscle–tendon units [14–16], and even microscopic MUs [17–20]. Such spatial information is also helpful in suppressing muscular cross-talks within channels so as to improve the signal–noise ratio. All these prominent features of applying the HD-sEMG techniques can be exploited and further promoted by the use of appropriate spatial filtering methods.

The spatial filtering technique can be employed to remove artifacts of HD-sEMG data and to retain useful information given the muscular activation heterogeneity. Its basic principle is to preserve the sources of interest and suppress unwanted components from signals [21–26]. Various matrix factorization algorithms [15, 16, 27–35] relied on different criteria concerning inherent structure of the input multivariate data. Among them, both principle component analysis (PCA) [27–29] and nonnegative matrix analysis (NMF) [14, 29, 33–35] algorithms have been commonly used due to their signal component separation capability, with successful applications in the field of decoding motor intentions including muscle strengths and patterns [27, 29, 36, 37]. The PCA is the most fundamental multivariate data analysis algorithm that can find a new set of projection directions called principle components (PCs) that explain the maximum variability of the original data. This projection allows specific manipulation of individual data components, and it is used to remove artifacts (i.e., common mode redundancy across multiple sEMG channels) and to retain useful information associated with specific projected data components. In addition, the NMF algorithm uses a non-negativity constraint that makes its outcomes physiologically meaningful [33]. Since NMF is commonly used to extract muscle synergies driven by the central nervous system to formulate muscle activities from multi-muscle sEMG recordings, it is more reasonable to extract and locate composing sources (i.e., muscle–tendon units [14, 19] or muscle belly regions [38, 39]) from heterogeneous muscle activation characterized by the HD-sEMG [34, 35]. Therefore, it facilitates to determine the task-related region within the coverage of the entire HD-sEMG array [14, 29]. In summary, relying on different criteria, both the PCA and NMF algorithms are expected to emphasize different signal components or to filter out different noises in the recorded HD-sEMG signals. Considering their own strengths and relative weaknesses, it is worth an attempt to use the combination of both algorithms due to their functional complementarity for motivating a better preprocessing outcomes.

It is hypothesized that spatial filtering can help to enhance and mine useful diagnostic-related information of HD-sEMG, thus improving diagnostic power of the sEMG examination. The purpose of this study is to verify this hypothesis by the means of testing it on the SCI data. Both the PCA and the NMF algorithms were selected for performing the spatial filtering due to their artifact removal and task-related region localization capabilities, respectively. Their combination was used as well by considering their functional complementarity. Clustering index (CI) analysis [40] was adopted as a representative and convenient approach for conducting the sEMG examination in this study due to its capability of revealing complex neuromuscular changes associated with the MU property alterations underlying paralyzed muscles. The CI analysis was originally designed to process routine single-channel sEMG signal for discriminating between neurogenic and myopathic diseases [40] and thereafter it has achieved great success in non-invasive diagnosis of amyotrophic lateral sclerosis [41], spinal and bulbar muscular atrophy [42] and stroke [43]. Our work not only applies the sEMG CI examination to the SCI data to investigate neuromuscular changes, but also proves the benefit of applying spatial filtering to HD-sEMG data for improving CI diagnostic power. Meanwhile, it evolves a series of PCA-based and NMF-based spatial filtering methods, which help to form a standard pipeline for HD-sEMG preprocessing before its clinical applications including diagnosis of neuromuscular changes.

## Methods

### Subjects

Nine subjects with incomplete cervical SCI (S1-S9, ASIA C or D) were recruited from the Clinical Neuroscience Research Registry at the Chicago Rehabilitation Institute (Chicago, IL, USA). Demographic and clinical measures for the subjects with SCI are summarized in Table 1. In addition, thirteen neurologically intact subjects and (C1–C13) without any neuromuscular disorder or injury also participate into the experiments.

**Table 1:** Physical characteristic of subjects with spinal cord injury

ID #	Gender	Age (years)	Level of injury	ASIA Class	Years since injury
S1	F	37	C7	C	4
S2	M	57	C7	D	17
S3	M	32	C6	C	11
S4	F	42	C4	D	7
S5	M	62	C4	D	8
S6	M	44	C5	C	11
S7	M	54	C4	D	12
S8	M	38	C7	C	12
S9	F	50	C8	D	7

### Experiments

**Fig. 1:**
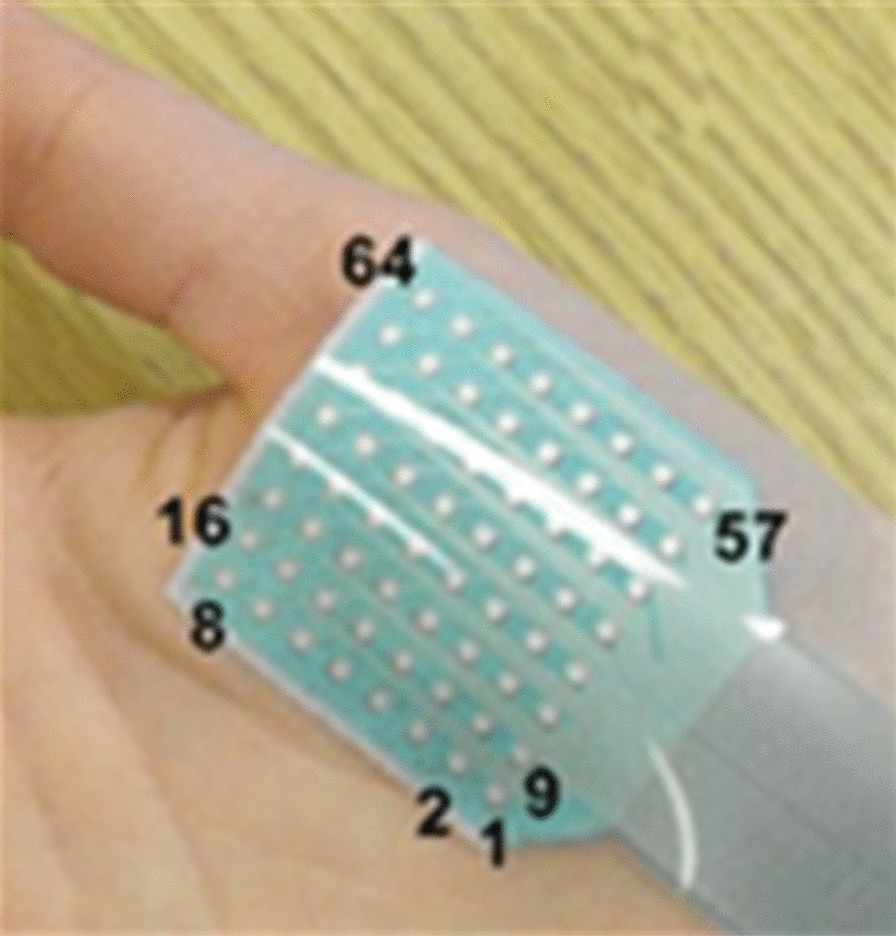
The flexible electrode array of 64 monopolar electrodes arranged in an 8 × 8 grid formation

The abductor pollicis brevis (APB) muscle was examined in this study. It is the largest and superficial muscle within the thenar muscle group on the palm with a distinct and simple function of thumb abduction. This distal muscle on the hand is representative for reflecting motor impairments [9, 10, 41, 43]. These features make it convenient to be examined following clinical routine. The data collection experiments were conducted on both sides of the subjects with SCI respectively, in a random order. The same experimental procedure was just applied to a randomly selected side of each control subject. On this basis, all the tested muscles can be categorized into three groups: the muscles on the left side of subjects with SCI (denoted as SCI-left group), the muscles on the right side of subjects with SCI (denoted as SCI-right group) and the control muscles from the neurologically intact subjects (denoted as control group). A flexible electrode array consisting of 64 electrodes in an 8 × 8 grid formation was used to target at the examined APB muscles, as shown in Fig. 1. Each electrode had a round recording probe in a diameter of 1.2 mm, and the center-by-center distance was 4 mm between two consecutive electrodes. The surface EMG signals were collected by a Refa128 EMG Recording System (TMS International BV, Enschede, The Netherlands) in 64 recording channels as a result of mono-polar configuration. The sampling rate was set at 2 kHz per channel. There is another round electrode (Dermatrode; American Imex, Irvine, CA, USA) placed over olecranon of the tested arm as the ground reference for the recording system.

The experiment was carried out in a quiet room in order to reduce the impact of the environmental noises. During the experiment, subjects were seated in a comfortable mobile chair. Their tested arm was bent approximately 90° and was placed on a height-adjustable desk. In the beginning of the experiment, the subject was encouraged to perform three maximal voluntary contractions (MVCs). The maximum value of these trials determined by monitoring the EMG amplitude was taken as a valid MVC. Then the subject was asked to generate an isometric contraction by abducting the thumb with increasingly graded force levels, roughly corresponding to 10%, 30%, 50%, 70%, submaximal (90%) and maximal voluntary contraction (MVC) in terms of the MVC percentage via the EMG amplitude. The subject was encouraged to remain at least 3 s as stable as possible for each contraction level. Sufficient rest was also allowed to avoid muscle fatigue between two consecutive trials.

**Fig. 2:**
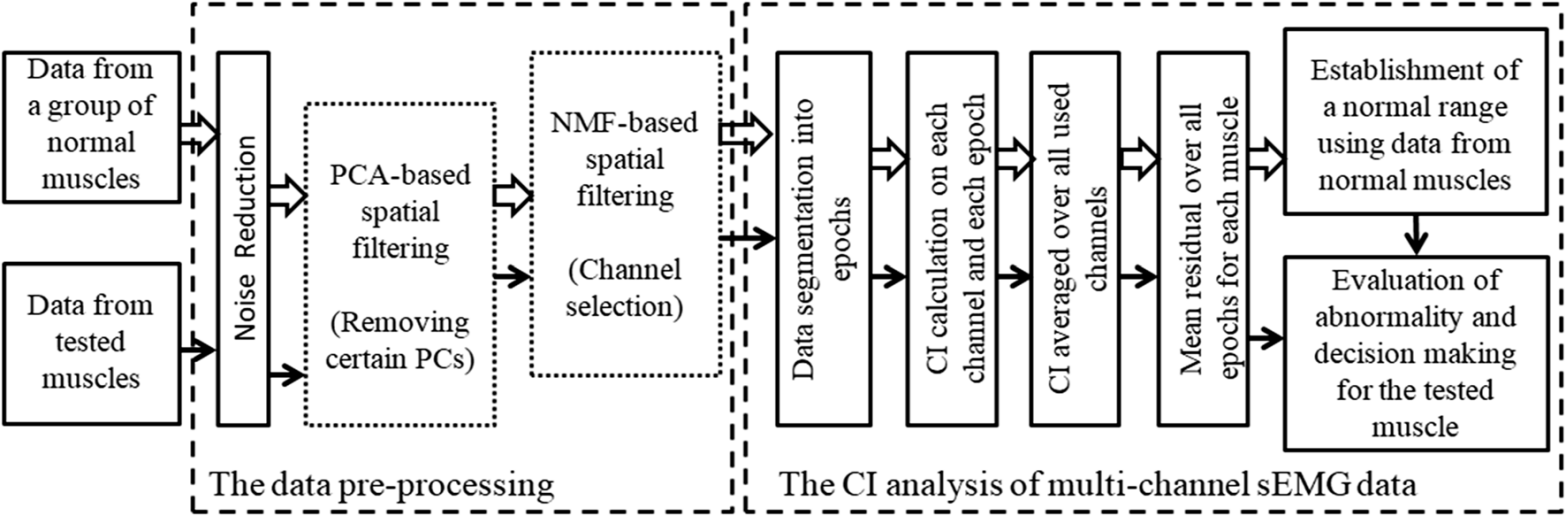
Block diagram of the framework for examining neuromuscular changes using the CI method and the spatial filtering analyses

The raw HD-sEMG data collected from the APB muscles on both sides of subjects with SCI and on a random side of control subjects were imported to the MATLAB (Version R2016a, MathWorks, Natick, MA, USA) software for analysis. Figure 2 shows the entire framework for examining neuromuscular changes through spatial filtering analysis and subsequent CI analysis of the HD-sEMG data, with more details described as follows.

### Signal preprocessing

A fourth-order Butterworth band-pass filter at 20–500 Hz was applied to eliminate potential low-frequency noises (e.g., motion artifacts) and high-frequency interferences. Then, a set of second-order notch filters were used to remove the 50-Hz power line interference and its harmonics. Subsequently, the spatial filtering methods could be applied to the HD-sEMG data.

#### Spatial filtering using PCA

In the PCA algorithm, the calculation of PCs is realized by diagonalization of the covariance matrix of data. The relevance of the PCs can be ranked in terms of the eigenvalues and reflect its contribution to the data in terms of variance for every PC. Suppose that the original signal ${M}_{0}^{\mathrm{m}\times \mathrm{t}}$ is in a form of m rows and t columns, where m represents the number of channels (64 in this study), and t is the sEMG signal sampling points. The transpose matrix $M$ of observation matrix ${M}_{0}^{\mathrm{m}\times \mathrm{t}}$ can be decomposed as Eq. (1).

1 $$M = UDV^{T} ,$$

where $D=diag({\lambda }_{1},\ldots ,{\lambda }_{\mathrm{m}})$ is the m × m diagonal matrix with ordered eigenvalues, the columns of the t × m orthogonal matrix *U* are the corresponding eigenvectors, and the m × m orthogonal matrix V satisfies $U{U}^{T}={V}^{T}V=$1. Thus the eigenvalue $\lambda$ and the eigenvector $U$ were calculated to decompose 64-channel sEMG signals into 64 PCs ${[U}_{1},{U}_{2},\ldots ,{U}_{64}]$ corresponding to their eigenvalues (${\lambda }_{1}\ge {\lambda }_{2}\ge \cdots \ge {\lambda }_{64}>0$) in a descending order. The eigenvectors describe the spatial distribution of the projected EMG over the grid that evolves in time. It has been supposed that the high eigenvalues of the first two components carry a substantial amount of redundant information (i.e., common mode) among multiple channels, and the components corresponding to the smallest few eigenvalues contain noises unrelated to EMG signals [28, 29, 44]. Therefore, it is necessary to remove these components. The PCs with the largest two eigenvalues and a number of smallest eigenvalues were intentionally selected and discarded, and therefore the remaining PCs were used to reconstruct the filtered signal ${M}^{^{\prime}}$. An sensitivity analysis was conducted to determine the number of components with the smallest eigenvalues to be removed, according to the diagnostic performance.

#### Spatial filtering using NMF

The NMF algorithm is formulated as a solution to a minimization problem with nonnegative bound constraints [45]. In this study, the multi-channel normalized and rectified sEMG matrix $X\in {R}^{m\times t}$ (m channels, t samples) was decomposed into two non-negative matrices $W\in {R}^{m\times s}$ and $C\in {R}^{s\times t}$ (where s < m) by the NMF algorithm according to Eq. (2):

2 $${X}^{m\times t}={W}^{m\times s}{C}^{s\times t}.$$

The matrix W can be interpreted as a number *s* of activation patterns, while the matrix C represents their corresponding time-varying activation coefficients. For processing the HD-sEMG data recorded on an entire muscle, each column of *W* here represents a spatially correlated activation pattern over the *m*-channel electrode array, anatomically reflecting the localization of a specific signal source. Thus, each row of the matrix C here specifies how an activation pattern is modulated during the task performance. The variable *s* varies from 1 to *m*, representing the number of activation patterns. In practice, the average variability accounted for (VAF, ranges from 0 to 1) between the original matrix ($X$) and the reconstructed one (${X}^{r}$) was calculated to determine the least number of activation patterns.

3 $${\text{VAF}} = 1 - \frac{{\mathop \sum \nolimits_{i,j} \left( {X - X^{r} } \right)_{ij}^{2} }}{{\mathop \sum \nolimits_{i,j} X_{ij}^{2} }}.$$

The value of VAF should be as large as possible while retaining original data information. Previous studies explained the success of reconstruction when the VAF reaches to 95% [29, 46]. Similarly, the number *s* was searched within a range between 0 and 6 and determined in this study when the VAF value was beyond 95%.

For each activation pattern, its corresponding time-varying coefficients were summed up and defined as activation intensity, according to Eq. (4):

4 $$Intensity\left( i \right) = \mathop \sum \limits_{j = 1}^{t} C_{i} \left( j \right),$$

where ${C}_{i}\left(j\right)$ represents the time-varying coefficients for the *i*th activation pattern, and *t* indicates the length of samples indexed by *j*. Among *s* activation patterns, the one corresponding to the highest *Intensity* value was considered to be the major activation pattern. In the major activation pattern, a number of channels with top-ranked weighting factors were considered to form a major activation region, and the channels of the input sEMG in such major activation region were just selected for further processing.

It was very critical to determine the number of channels to be selected in the major activation region. Similarly, a sensitivity analysis was conducted in this study by varying the number from 14 to 18, and this number was appropriately set according to the optimal diagnostic performance.

### Data segmentation and CI analysis

The data (in a form of multiple channels regardless of whether they were spatially filtered or not) presented several segments of sEMG activities according to the experiment protocol. The onset and offset of each sEMG activity burst can be easily determined. For CI analysis, a series of 1-s epochs (equivalent to 2000 sample points at the sampling rate of 2000 Hz) need to be segmented from the EMG activity. Since actual muscle force was not recorded in this study, the generally observed sEMG amplitude intensities were used to roughly estimate the muscle force fluctuations. The force was considered to be relatively stable when the sEMG intensity remained at a consistent level. Otherwise, the corresponding epochs would be discarded especially at the switch of two consecutive force levels. Thus, the number of epochs in a multi-channel form ranged from 20 to 40 over all force levels for each of all subjects.

CI is a non-invasive quantitative method for analyzing uneven distribution and cluster of the processed signal to different neurogenic and myopathic changes [43]. To calculate CI, the signal in each epoch was divided into several non-overlapping consecutive windows in length of 15 ms, which is regarded to approximately cover an individual MUAP [40]. Suppose that there are K windows in total derived in an epoch and Ai was the area of each window. The differential sequences between every consecutive area value (${\mathrm{DA}}_{i}$), between every second window (${\mathrm{DB}}_{i}$) and between every third window (${\mathrm{DC}}_{i}$) can be calculated. Then the CI of each epoch is defined as:

5 $$\mathrm{CI}=\left\{\sum\limits_{i=1}^{K-1}{DA}_{i}^{2}+\sum\limits_{i=1}^{K-2}{DB}_{i}^{2}+\sum\limits_{i=1}^{K-3}{DC}_{i}^{2}\right\}/\left(6\bullet \sum\limits_{i=1}^{K}{A}_{i}\right).$$

The CI has a value between 0 and 1, and a relatively high value represents a highly clustered signal, appearing with isolated large action potentials. If the EMG epoch carries multiple channels (it is always the case in this study), the average CI value was calculated from all chosen channels. While the values of CI depended on the contraction level: the increase in contraction levels resulted in a lower value, the area of each epoch, that is the area of all windows, was used to estimate the muscle contraction level [40]. It had been proven to be linearly related to CI values using double logarithmic scale [47]. Hereafter, the average areas for all selected channels on each epoch were calculated. For each analysis epoch on the same muscle, two values were obtained: a mean log (area) and a mean log (CI), which were expressed as a point in the CI-area plot. The points derived from the analysis epochs can be scattered to form a cloud over the CI-area plane.

The quantification of the normal data reference in the CI-area plot is the prerequisite to subsequent diagnosis. To establish the distribution of the normal cloud, a linear regression analysis was performed on all analysis epochs (1 $\le \mathrm{area}\le 100\mathrm{\mu V}\cdot \mathrm{sec}$)from the healthy subjects was performed for both log(CI) and log(area). For each epoch, the deviation of the log (CI) scale from the linear regression line was calculated. Then these deviation values were averaged to obtain a mean residual (denoted as Rm), which can be used to assess the presence of abnormality for each subject. The mean u and standard deviation ($\upsigma$) of the $Rm$ values on the two sides of all the subjects were calculated and then a Z score was computed as the final quantify indicator for the evaluation.

6 $$\begin{array}{*{20}c} {Z = \frac{Rm - \mu }{\sigma }.} \\ \end{array}$$

A Z score between $\pm 2.5$ was defined as abnormal. A tested muscle with a Z score higher than + 2.5 indicated neurogenic changes while a Z score lower than − 2.5 was diagnosed as being myopathic changes.

### Performance evaluation and statistical analysis

To evaluate the effect of spatial filtering in HD-sEMG-based diagnosis, the PCA-based method, the NMF-based method and their combination termed the PCA-NMF-based method were applied for spatial filtering of the HD-sEMG signals, with comparison of the original HD-sEMG without any spatial filtering approach. These three spatial filtering methods were shown together in Fig. 2, where the PCA-based method and the NMF-based method can be used optionally and separately (each in a dotted block representing an optional procedure). In the PCA-NMF-based method, both the PCA-based method and the NMF-based method were implemented sequentially. Regardless of whether the data were spatially filtered or not, the diagnostic analyses relied on application of the CI method to HD-sEMG data recorded from three muscle groups: SCI-left group, SCI-right group and control group.

Given a certain group of examined muscles, both the abnormal CI Z score increase and decrease can be simultaneously observed due to diversity of abnormality following SCI. This was the case in this study (as reported in the following “Results” section). For a specific muscle with certain abnormal changes, the greater Z score dispersion from the normal range was yielded by the examination approach (including the signal pre-processing method), the higher its diagnostic sensitivity became according to the CI calculation. Given a group of tested muscles expected to have changes in various type and degree, the variance of Z scores over this group could be used to evaluate the diagnostic sensitivity of the CI indicators, after different spatial filtering methods. Suppose the Z score variance was ${a}_{SCI}$ and ${a}_{control}$ calculated over a group of tested muscles from subjects with SCI and healthy controls, respectively. The value of ${a}_{control}$ of each tested muscle is 1 due to the defined normalization of diagnostic criteria in the CI method. The evaluation criterion abnormality discriminating index (ADI) was defined as a quantitative indicator of evaluating diagnostic power of the entire examination approach according to Eq. (7), which represents the sensitivity of identifying various types of neuromuscular changes from abnormal signals.

7 $$\begin{array}{*{20}c} {ADI = \frac{{a_{SCI} }}{{a_{control} }}.} \\ \end{array}$$

The ADI values were calculated respectively under different conditions. In this study, the condition was defined by the use of both the channel and the spatial filtering method for data analysis. A special condition was designed as a representative approach without any spatial filtering method for the comparison purpose, which simply averaged CI values over all used channels when HD-sEMG data were used. The higher an ADI value was yielded, the greater diagnostic sensitivity (to various alterations in the given subject population) the corresponding method had.

In order to verify the generally sequential consistency of individual muscles’ diagnostic outcomes, a series of linear regression analyses were performed on the CI Z scores derived from both the SCI-left and SCI-right groups, between any two different conditions. Two separate two-way repeated-measure ANOVAs were performed on the Z score, with the group (two levels: the control muscle group versus each of two muscle group with SCI, respectively) considered as the between-subject factor and the condition (four levels: the simply averaging approach and three spatial filtering methods) considered as the within-subject factor, to simultaneously examine their effect on the Z score group means. Another two-way repeated-measure ANOVA was performed, with both the side/group (two levels: the SCI-left group versus the SCI right group) and the condition (four levels) considered as within-subject factors. The level of statistical significance was set to *p* < 0.05 for all above analyses. All statistical analyses were completed using SPSS software (ver. 22.0, SPSS Inc. Chicago, IL, USA).

## Results

**Fig. 3:**
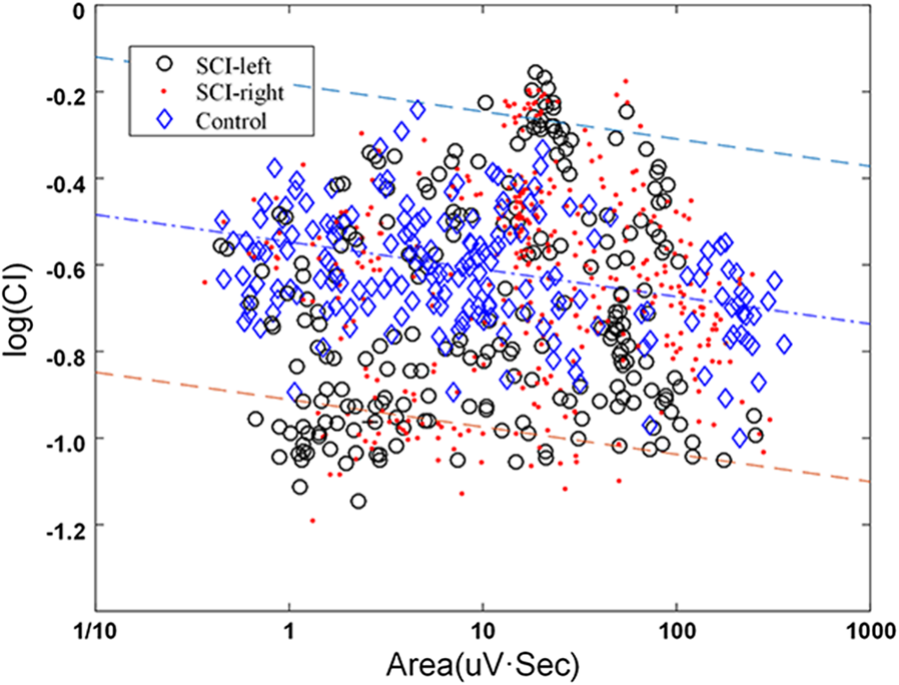
The CI-area plot of a deliberately channel presented in double logarithmic scale for the three groups: SCI-left group, SCI-right group and control group. The normal range (dotted line) is presented within + 2.5 of the standard error of the linear regression. The red dots and black circles represent the epochs of SCI-left group and SCI-right group were found to be outside the banding region

Figure 3 shows the resultant CI-area plot of the scattered data points from three muscle groups in the double logarithmic scale, when the data were only from a deliberately selected channel. For the normal cloud consisting of all data points from the control muscle group, the CI showed a decreasing trend as the contraction level increased. This was suitable for a linear regression analysis ($\mathrm{y}=-0.0631\mathrm{x}-0.5470$). Along the regression line, there is a banding region that can well characterize the distribution region of the normal cloud.

**Fig. 4:**
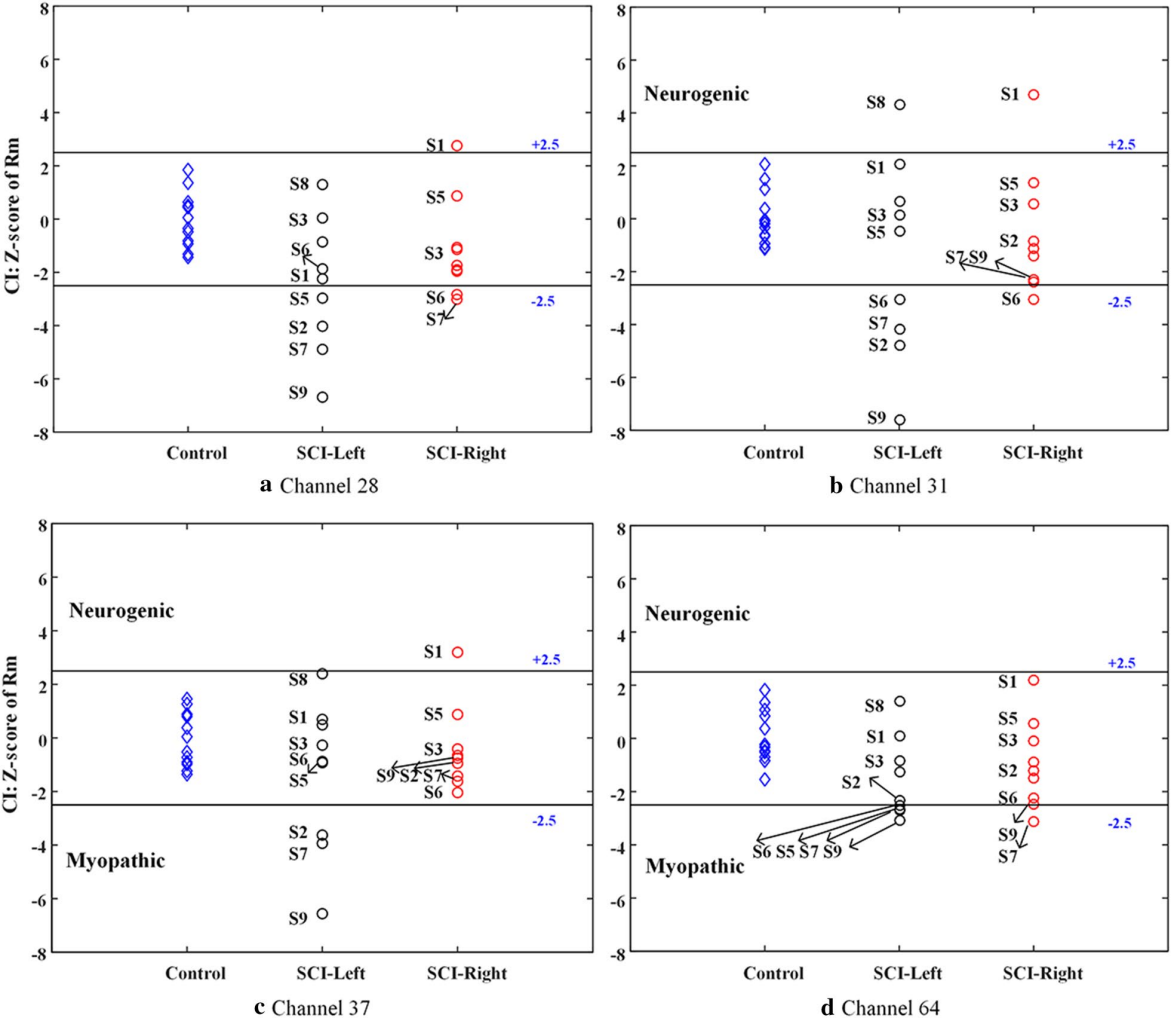
Z scores derived from four selected channels: **a** channel 28, **b** channel 31, **c** channel 37 and **d** channel 64. The Z scores from SCI-left group, SCI-right group and control group are shown separately. The normal range (solid line) is presented within ± 2.5

Figure 4 reports the CI Z scores when using data from each of four different channels (channel 28, channel 31, channel 37 and channel 64). It can be seen that although different CI Z scores and the corresponding diagnostic results obtained from different channels, the order of the CI Z scores was substantially the same. For example, for the left muscle of S8, data from channel 31 gave a diagnosis of abnormal increase, but the other three channels failed to report any abnormality. Using data from channel 37, the left muscle of S8 had a CI value that approximated to the upper limit of the normal boundary. It is surprisingly to find that the CI Z score value of S8 was always the highest on the SCI-left group. Similar observations can also be found in multiple cases such as S2, S7 and S9 on the SCI-left group and S1 on the SCI-right group. Further, for different channels, the data of the same group exhibited different degrees of dispersion. More abnormal diagnostic conclusions could be found for the group with high dispersion. It was confirmed that the ADI value was able to be used to judge the diagnostic power. Therefore, using four different channels had an impact on the diagnostic power, quantified by the ADI ranging from 2.3160 to 14.0252 for the SCI-left muscle group and 2.5341 to 5.7445 for the SCI-right group.

The PCA-based method yielded varying ADI values when the number of components to be removed corresponding to smallest eigenvalues was set from 0 to 6, respectively, as shown in Table 2. It can be found that although the resultant ADI values of both SCI-left and SCI-right groups were extremely closed, the highest ADI values were yielded by removing the 4 components with the smallest eigenvalues. Therefore, for implementing the PCA-based spatial filtering method, the number of PCs corresponding to the smallest eigenvalues to be removed was determined to be 4 in the following analysis throughout the study.

**Table 2:** The ADI values derived from removing different numbers of the components with the smallest eigenvalues, using the PCA-based filtering method

The number	ADI (SCI-left)	ADI (SCI-right)
0	3.1439	3.8592
1	3.1457	3.8628
2	3.1446	3.8654
3	3.1459	3.8681
**4**	**3.1488**	**3.8785**
5	3.1349	3.8663
6	3.1327	3.8632

The number of 4 leads to the maximal ADI values for both data groups (in bold)

**Fig. 5:**
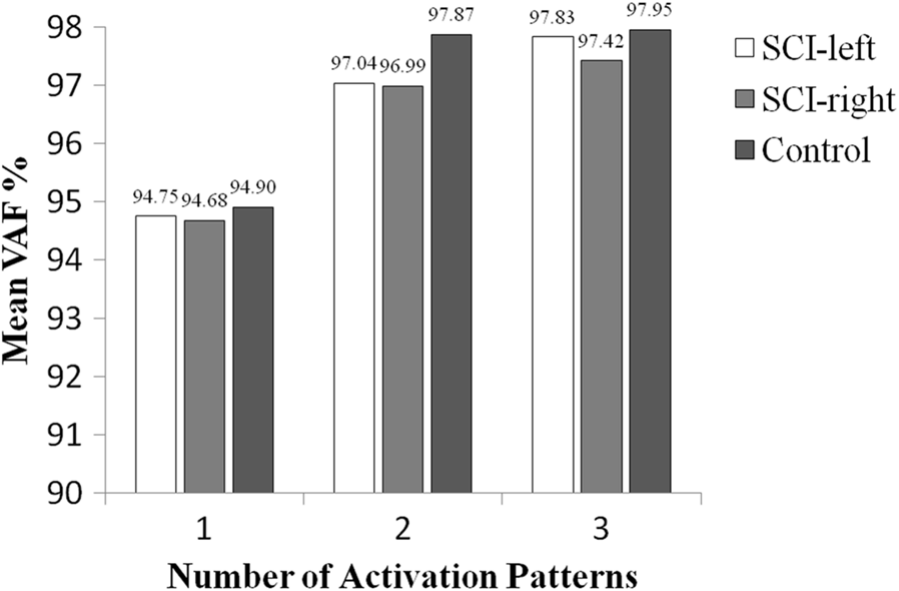
Mean VAF values averaged over all subjects in three groups (SCI-left, SCI-right, and Control group) when the number of activation patterns was set at 1, 2 and 3, respectively

Figure 5 shows the mean VAF values averaged over all muscles in three groups (SCI-left, SCI-right, and control group) when the number *s* of activation patterns was set at 1, 2 and 3, respectively. Evidently, the mean VAF exceeded over 95% when the number of patterns was increased from 1 to 2, and the addition of the 3rd activation pattern (the number is three) did not help to increase the VAF much. Therefore, the variable s was set to 2 in the NMF algorithm implementation.

Another sensitivity analysis was also performed to determine the number of selected channels optimally for implementing the NMF-based spatial filtering method, according to the diagnostic power. The ADI results were shown in Table 3 when the number ranged from 14 to 18. It can be observed that the number set at 16 led to the highest level of the ADI values for both muscle groups. Although the ADI was found to vary slightly with the number of selected channels, the actual filtering effect was insensitive to this number. Without loss of generality, this number was finally fixed to 16 (a quarter of the total channel number) in the following analyses when the NMF algorithm was involved for any spatial filtering (including the PCA-NMF-based method).

**Table 3:** The ADI values for both SCI muscle groups when the number of the selected channels ranged from 14 to 18 using the NMF-based spatial filtering method

The number	ADI (SCI-left)	ADI (SCI-right)
14	7.7595	3.5425
15	7.8055	3.5491
**16**	**8.0397**	**3.8033**
17	7.9549	3.6820
18	7.8013	3.8356

The number of 16 leads to the maximal ADI values for both data groups (in bold)

**Fig. 6:**
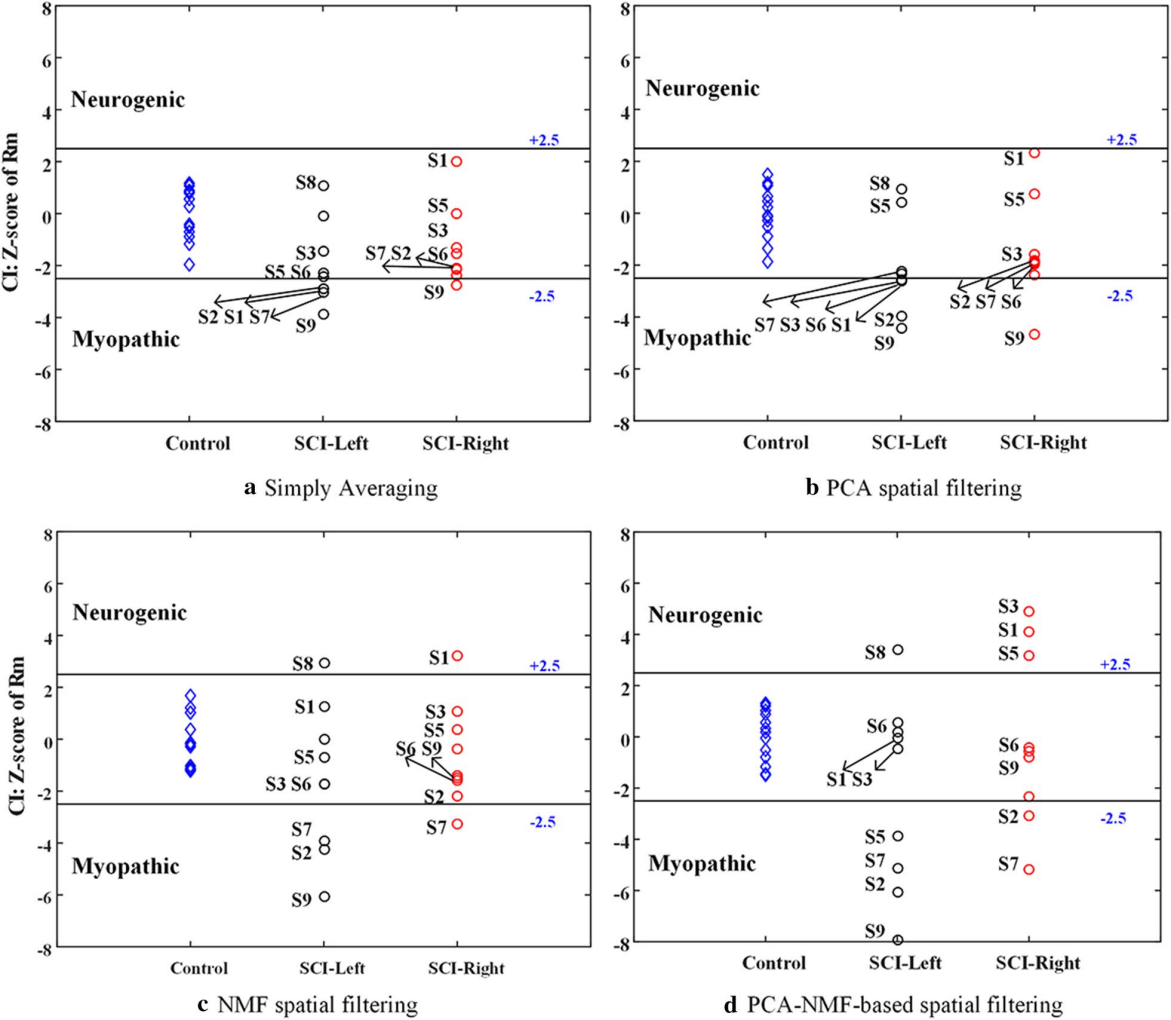
Z scores derived from four methods: **a** simply averaging, **b** PCA-based spatial filtering, **c** NMF-based spatial filtering, and **d** PCA-NMF-based spatial filtering. The Z scores from SCI-left group, SCI-right group and control group are shown separately. The normal range (solid line) is presented within ± 2.5

Figure 6 reports the resultant Z scores derived from multi-channel data for all three examined muscle groups when different spatial filtering methods were used respectively. The CI Z scores from the same group of muscles almost had a consistent order even comparing to that at any single channel in Fig. 4, regardless of whether the data were filtered by any spatial filtering method or not. When different spatial filtering methods were applied, however, the muscle with an abnormal decision had varied dispersions from the normal boundary. Specifically, the Z scores obtained by simply averaging CI values over all used channels were shown in Fig. 6 a. Although some muscles were reported to be abnormal, their Z scores were extremely close to the normal boundary, and the ADI value was reported to be 2.4936 for the SCI-left group and 2.1671 for the SCI-right group. Both ADI values were found to remain at a median level of the values derived from individual channels. After spatially filtering the HD-sEMG data using three methods, more abnormal Z scores are exhibited in Fig. 6b–d, and their dispersions from the normal boundary are relatively expanded as well. Thus, it is not accidentally that the ADI values were improved to 3.1488 for the SCI-left muscle group and 3.8785 for the SCI-right muscle group using the PCA-based method. Both values were 8.0397 and 3.8033 using the NMF-based method. Apparently, the PCA-NMF-based method presented the highest degree of Z score dispersion, and meanwhile it was able to reveal more abnormal muscles. For example, the use of the PCA-NMF-based method successfully produced abnormally high Z scores for the S3 and S5 on the SCI-right group, whereas the use of any other spatial filtering method or any single channel failed to reveal any abnormality. Finally, the ADI values yielded by the PCA-NMF-based method reached to 13.9157 for the SCI-left group and 11.7014 for the SCI-right group, which approximated into or even exceeded the maximal level of the ADI values derived from individual channels.

The linear regression analyses reported strong correlations (R2 from 0.80 to 0.93) between the CI Z scores made by any spatial filtering method and the simply averaging approach (i.e., no spatial filtering method), and estimates of coefficients were all statistically significant (*p* < 0.001). The ANOVAs revealed no significant main effect of the group and no significant difference in group means of the CI Z score between any muscle group following SCI and the control muscle group, or no significant main effect of the spatial filtering method (*p* = 0.855) on the CI Z score. However, significant difference was found between the SCI-left muscle group and the SCI-right muscle groups (*p* < 0.05).

The final ADI values derived from original sEMG data at four individual channels and spatially filtered HD-sEMG data via different spatial filtering methods were summarized in Table 4. It showed that the ADI values of different channels might be quite different. Comparing to the simply averaging HD-sEMG data without any spatial filtering, the PCA and NMF spatial filtering methods had larger ADI values on both the SCI-left and the SCI-right groups. Moreover, the PCA-NMF-based spatial filtering method outperformed other methods by yielding almost the largest ADI values on both groups of muscles following SCI.

**Table 4:** The ADI values derived from different methods

Condition	Left	Right
CH28	6.2292	3.4041
CH31	14.0252	5.7445
CH37	7.7690	2.5341
CH64	2.3160	2.7312
Simply averaging	2.4936	2.1671
PCA-based	3.1488	3.8785
NMF-based	8.0397	3.8033
PCA-NMF-based	13.9157	11.7014

## Discussion

This study presents three spatial filtering methods for preprocessing HD-sEMG data to enhance the power of assessing neuromuscular abnormalities following SCI. The primary findings of the current study include: (1) the complex neuromuscular changes following SCI were revealed by the CI analysis of conventional single-channel sEMG, and the diagnostic power could be characterized by the variance of Z scores derived from a group of subjects (as shown in Fig. 4); (2) the diagnostic power was found to vary across positions of individual channels for recording sEMG data (see Fig. 4 and Table 4), and it only remained at a median level when all the CI values derived from all recording channels were simply averaged (see Fig. 6); (3) The application of PCA-based filtering method or NMF-based filtering method helped to improve the diagnostic power significantly, and the method with their combination outperformed any single method in terms of diagnostic power (as shown in Fig. 6 and Table 4); (4) A subject with SCI might have pathological changes on both sides of muscles in different types and at different degrees (see Fig. 6).

### MU alterations following SCI evaluated by CI method

The CI method was traditionally used for single-channel sEMG analysis. Regardless of any channel (within the array) used for analysis, it can be observed from Fig. 4 that each examined muscles (in both SCI-left and SCI-right groups) tended to have a consistent decision. Furthermore, all muscles from subjects with SCI exhibited three different CI patterns including normal and abnormal increase and decrease of the CI indicator.

Four muscles of the SCI subjects had an abnormal CI Z score increase indicating neurogenic changes. These changes can be attributed to loss of MUs and subsequent reinnervation of denervated muscle fibers. The MU loss may take place after gray matter is destroyed at and near the lesion epicenter and it can lead to a decrease in the number of activable MUs and denervation of muscle fibers [3]. Complete denervation due to motoneuron degeneration eliminates voluntary control of the affected muscle fibers. Subsequently, the surviving MUs tend to undergo adaptive changes, such as muscle fiber reinnervation for a functional supplement, thus contributing to an abnormal enlargement of their structures [48]. These enlarged MUs lead to abnormal MUAPs with large amplitude and multiple phases, overlying into scattered and isolated EMG signals. In addition, after chronic (> 1 year) SCI, MU properties of human hand muscles shifted towards decreased firing rate and increased firing synchronization [49]. Simultaneously, other altered MU control properties including the compression of MU recruitment threshold and the supplementary recruitment of enlarged MUs during muscle contraction might also lead to an abnormal increase of CI [2, 50].

Eleven muscles had abnormally lower Z scores indicating myopathic changes, which could be related to muscle fiber disuse atrophy. Atrophied and angular muscle fibers could lead to partial denervation, which can be indicated by intramuscular motor axon sprouting, an important compensatory mechanism for recovery of muscle innervation after death of some motoneurons [51]. A selective degeneration of the relatively larger and superficial MUs may be another reason. Thus the induced flatter and denser surface EMG signals would make decreased CI values [52].

The resultant Z scores of remaining muscles were located within the normal range. However, substantial muscle weakness was also found in these muscles. Their paralyses are likely to be attributed to a deficit of descending central drive as a result of the severance of central nervous system axons and demyelination of central or peripheral axons, while the affected muscles still function more or less normally [53]. Although the number of activable MUs drops, their recruitment and control property remains similar to those of healthy controls. Thus, these muscles could only deliver a fraction of the normal voluntary drive, leading to corresponding muscle weakness [42]. Another possible explanation for the distribution in the “normal range” might be a combined or cancelled effect of both neurogenic and myopathic processes [54]. Moreover, the effect of injury on the lesion spinal cord segment and denervation of muscle fibers might be contributors to muscular weakness [49]. Therefore, the experimentally observed CI variations in paralytic muscles can be viewed as the overall or collective effects of these different factors [47].

As a result, the experimentally observed CI abnormality consists of two patterns, which lead to CI deviation in two different directions, respectively. Therefore, a pooled analysis of a group of paralyzed muscles following SCI can allow their CI indicators to spread from the centered normal range, indicating that there are complex neuromuscular changes following SCI. This phenomenon explains why there was no significant difference in group means of CI Z scores in the ANOVAs, and therefore the ADI was more appropriate to characterize the diagnostic power of the CI method in this study.

### Examination with HD-sEMG recording

Given the HD-sEMG recording, varied distributions of the CI Z scores were observed and thus different diagnostic decisions were made when data from different channels were used (see Fig. 4). Thus, it also directly led to different diagnostic power quantified by the ADI value. This confirms our previous assumption that the important diagnostic information is likely to be derived from some local regions of the electrode array due to the heterogeneity of the targeted muscle. This finding also suggest a risk of electrode placement when applying the routine single-channel sEMG recording, while its clinical application has been increasingly investigated toward noninvasive examination. Depending on the placement of the sEMG electrode (targeting at a local region of the examined muscle), the CI diagnostic decision varied a lot, probably leading to controversial results. This may dramatically impact the usability of the sEMG examination. In addition, we also found that the channel with the highest diagnostic power was not always located at the center of the array or over the position of main muscle belly. Such a finding further indicates the importance of electrode placement since the channel location yielding the most diagnostic power is usually uncertain solely relying on anatomical knowledge.

Taking the averaged CI value over HD-sEMG channels is the most straightforward method to extract the global information of muscle activity and eliminate the influence of the electrode position. However, unsurprisingly, we found that the ADI diagnostic power obtained in this way was only at the median level of those using individual channels. This shows that simply averaging may smooth or cancel the useful diagnostic information present in the local channels, which is detrimental to revealing specific abnormalities in individual muscles. In this case, the resultant CI indicator reflected poor understanding of underlying pathological muscle changes.

### The advantages of spatial filtering

After the determination of appropriate settings towards performance optimization (see Fig. 5, Tables 2, 3), the improved performance yielded by the use of either PCA-based or NMF-based spatial filtering method in terms of increased ADI value can demonstrate the efficiency of applying the spatial filters. Furthermore, the strong correlations revealed by the regression analyses between individual muscles’ Z scores derived from any spatial filtering method and the simply averaging approach indicate consistency of their diagnostic decisions (they are able to produce or tend to produce the same type of abnormality for specific muscles). These findings suggest that the use of spatial filters enhances the sensitivity of HD-sEMG CI indicator to various neuromuscular changes.

The PCA-based filtering method was designed to deliberately remove PCs representing homogeneously changing and common features, and detail PCs representing high-frequency noise and cross-talk [29, 44]. Such processing helps to enhance regional difference of the signal and is considered to be the main reason for diagnostic power improvement by the PCA-based spatial filtering method. Unlike the PCA-based method, the NMF-based method is equivalent to a channel-selection method by extracting distinguishable muscle activation patterns based on muscle heterogeneity [50]. The method is actually a dimensionality reduction processing that locates and highlights the main areas within the HD-sEMG array contributing into muscle activities.

According to their calculation principles, both algorithms were regarded to emphasize different aspects of information convoyed in the raw HD-sEMG data. As a result of their complementary effect, it is easy to explain that their combination can further improve the diagnostic power in comparison to sole use of the PCA-based or the NMF-based method.

The HD electrode array physically covers the examined muscles, providing a wealth of spatial information in a large area. Direct averaging these channels is not satisfactory for diagnostic improvement. By contrast, spatial filtering methods evidently improve the performance of HD-sEMG examination, approximating to the maximal level of diagnostic powers when individual channels are used. Thus, the use of spatial filtering helps to highlight and refine useful diagnostic information associated with heterogeneity of the muscle activation, and provides a necessary and convenient approach to pre-process HD-sEMG data for further examination of neuromuscular changes. In addition to enhancing diagnostic power, this can also minimize the potential influence of electrode placement. The spatial filtering of HD-sEMG can facilitate sEMG examination, indicating the ease of reflecting potential abnormality in certain muscles by the means of a noninvasive approach.

It is worth noting that the presented spatial filtering methods involve an unsupervised matrix factorization algorithm without any association with the diseased or the diagnostic information (no label is required). The filtering approach was conducted independently on the data recording trial or the examined muscle. Thus, it is straightforward to apply the proposed method to data from any given muscle to be examined, and the filtering outcome is supposed to rely on the structural nature of its HD-sEMG data. All these features confirm a good generalization of the proposed spatial filtering method. This is also a prerequisite to its involvement in a standard pipeline for preprocessing the HD-sEMG signals towards various applications.

### Difference between two sides of the subject with SCI

Since the CI analysis performed the examination of individual muscles, it is straightforward to compare the neuromuscular changes in muscles on both side muscles of a subject. It was observed that the two side muscles of some SCI subjects showed different CI decisions. For example, the left muscles of S1, S3 and S5 were diagnosed as being ‘myopathy’ while their contralateral muscles were diagnosed as being ‘neurogenic’ changes. This may be attributed to the asynchrony of the left and right muscles. When the physiological balance is broken following SCI, it always overcorrects so that the left and right muscles may present different types of neuromuscular changes at different degrees.

### Limitations of the current work and future expectations

This paper just focuses on the application of spatial filtering and presents only three types of common spatial filtering methods. The use of the CI method has also limited performance in examining specific MU property alterations. More advanced methods including sophisticated spatial filtering methods and more sufficient diagnostic indicators can be developed for improved performance. In addition, the sample size used in this study is relatively small. It is sufficient only for technically confirming the benefit of using the spatial filtering methods in HD-sEMG diagnosis. Although the CI method would be applicable for examining muscles with diverse impairments at any degrees, the sEMG diagnostic approach requires more or less voluntary contraction ability of the examined muscle to emit sufficient sEMG activities. This is the main reason for recruiting subjects with incomplete SCI preserving certain hand functions in this study. The small sample size limits clinical significance of the current study. In order to establish diagnostic criteria and reveal neural or muscular pathology, a big sample size from a large population of subjects with different impairments is demanded. These will remain our future work.

## Conclusion

This paper examined the feasibility of performing spatial filtering methods using the PCA algorithm, the NMF algorithm and their combination, for enhancing HD-sEMG examination of neuromuscular changes. The experimental results demonstrated that spatial filtering of HD-sEMG can help to improve diagnostic power of CI method with respect to that with no spatial filtering, and that the combined PCA-NMF-based spatial filtering method yielded the highest diagnostic power in identifying complex neuromuscular diseases following SCI. The proposed method facilitates HD-sEMG examination of neuromuscular changes and it helps to develop a standard pipeline for pre-processing the HD-sEMG data towards practical and meaningful applications.

## Data Availability

The datasets of the experiments in the current study are available from the first author on request.

## Abbreviations

HD: High-density
sEMG: Surface electromyogram
MUAP: Motor unit action potential
PCA: Principle component analysis
NMF: Non-negative matrix factorization
CI: Clustering index
SCI: Spinal cord injury
MU: Motor unit
APB: Abductor pollicis brevis
MVC: Maximal voluntary contraction
PC: Principal component
ADI: Abnormality discriminating index
